# First draft genome of *Thecaphora frezii*, causal agent of peanut smut disease

**DOI:** 10.1186/s12863-023-01113-w

**Published:** 2023-02-16

**Authors:** Renee S. Arias, Cinthia Conforto, Valerie A. Orner, Edgardo J. Carloni, Juan H. Soave, Alicia N. Massa, Marshall C. Lamb, Nelson Bernardi-Lima, Alejandro M. Rago

**Affiliations:** 1 USDA-ARS National Peanut Research Laboratory (NPRL), Dawson, GA USA; 2 Instituto de Patología Vegetal, Centro de Investigaciones Agropecuarias, Instituto Nacional de Tecnología Agropecuaria, IPAVE-CIAP-INTA, X5020 Córdoba, Argentina; 3 Unidad de Fitopatología y Modelización Agrícola, Consejo Nacional de Investigaciones Científicas y Técnicas, UFYMA-CONICET, X5020 Córdoba, Argentina; 4 Instituto de Fisiología y Recursos Genéticos Vegetales, Centro de Investigaciones Agropecuarias, Instituto Nacional de Tecnología Agropecuaria, IFRG-CIAP-INTA, X5020 Córdoba, Argentina; 5 El Carmen S.A., General Cabrera, Córdoba, Argentina; 6 Centro de Investigaciones Agropecuarias - INTA, Córdoba, Argentina

**Keywords:** PacBio, Genome, Pathogen, Groundnut, Smut disease, Fungi

## Abstract

**Objectives:**

The fungal pathogen *Thecaphora frezii* Carranza & Lindquist causes peanut smut, a severe disease currently endemic in Argentina. To study the ecology of *T. frezii* and to understand the mechanisms of smut resistance in peanut plants, it is crucial to know the genetics of this pathogen. The objective of this work was to isolate the pathogen and generate the first draft genome of *T. frezii* that will be the basis for analyzing its potential genetic diversity and its interaction with peanut cultivars. Our research group is working to identify peanut germplasm with smut resistance and to understand the genetics of the pathogen. Knowing the genome of *T. frezii* will help analyze potential variants of this pathogen and contribute to develop enhanced peanut germplasm with broader and long-lasting resistance.

**Data description:**

*Thecaphora frezii* isolate IPAVE 0401 (here referred as T.f.B7) was obtained from a single hyphal-tip culture, its DNA was sequenced using Pacific Biosciences Sequel II (PacBio) and Illumina NovaSeq6000 (Nova). Data from both sequencing platforms were combined and the de novo assembling estimated a 29.3 Mb genome size. Completeness of the genome examined using Benchmarking Universal Single-Copy Orthologs (BUSCO) showed the assembly had 84.6% of the 758 genes in fungi_odb10.

## Objective

Peanut smut disease converts the peanut seed into a brown/black powder of fungal teliospores, example shown in Data File 1, Table 1 [1]. The disease is currently endemic to Argentina [2], where the pathogen *Thecaphora frezii* [1] has been reported to cause up to 51% disease incidence [3] and crop losses up to 35% [2, 4]. Global trade could potentially spread the disease to other areas. For example, United States is a major exporter of peanuts [5], but it also imports peanuts from other countries [6] including Argentina [7]. Scientists from the U.S. and Argentina are working in collaboration to better understand the disease and to identify resistant peanut germplasm [8–11]. Part of that effort is to know the genetics of the pathogen. Previously, we have sequenced the 123 kb mitochondrial genome of *T. frezii* based on DNA obtained from teliospores [12]. Other than that, the National Center for Biotechnology Information (NCBI) has only 170 entries for this species, those sequences are under 2,500 bp, and more than half correspond to microsatellites and ribosomal RNA. We have found that working with teliospores had its own constraints, thus, the current work aimed to sequence the genome of a hyphal tip culture of this fungus. The isolate referred as T.f.B7 was obtained from peanuts in Argentina and is now stored in the IPAVE culture collection as IPAVE 0401, both, the hyphal tip culture and the original teliospores are kept in the collection. Overall, BLAST analysis performed on assembled *T. frezii* contigs showed highest similarity (~ 78%) to *Anthracocystis flocculosa* (Syn. *Pseudozyma flocculosa*), followed by (~ 76%) similarity to *Sporisorium* spp. and lower similarity (~ 73%) to *Ustilago* spp. Given the limited information available about this pathogen, the data are very useful.

**Table 1:** Overview of data files/data sets

Label	Name of data file/data set	File types (File extension)	Data repository and identifier (DOI or accession number)
Data file 1	Peanut smut in shell *Thecaphora*_*frezii*_culture	Tag Image File Format (.tiff)	https://doi.org/10.7910/DVN/EOYLUX [1] at Harvard Dataverse
Data file 2	QC_140bp	Excel (.xlsx)	https://doi.org/10.7910/DVN/I338KA [13] at Harvard Dataverse
Data file 3	Contig_Length_&_Cumulative Length_TF	Portable Document Format (.PDF)	https://doi.org/10.7910/DVN/SOFHRR [14] at Harvard Dataverse
Data set 1	B7_S113_L004_R1_001 B7_S113_L004_R2_001	Illumina NovaSeq 6000 data; Fastq file (fastq.gz)	SRR18840655 [15] at NCBI GenBank Database
Data set 2	M64069_211005_200549	PacBio, Binary alignment map (.BAM)	SRR18837637 [16] at NCBI GenBank Database
Data set 3	*T. frezii* Genome Assembly: 7166_Contigs_TF_B7_Genome	Fasta (.fasta)	JALNIF000000000 [17] at NCBI GeneBank Database

### Data description

*Thecaphora frezii* isolate IPAVE 0401 (here referred as T.f.B7), was obtained as a hyphal culture from teliospores of a smut-affected peanut plant collected in 2018 from Hernando city, in Tercero Arriba, Cordoba (32° 24′ 30.5028″ S, 63° 42′ 18.9468″ W). Genomic DNA was extracted from T.f.B7, a culture expected to be haploid monokaryotic as shown by HCL-Giemsa and Propidium iodide nuclear staining, Data File 1, Table 1 [1], and was sequenced as paired end 150 base pairs (bp) using Illumina NovaSeq6000 which resulted in 114,541,382 reads, Data set 1, Table 1 [15]. After trimming for potential presence of adapters and removal of sequences shorter than 140 bp, 112,980,831 clean reads were available. A summary of read length and quality is listed in Data File 2, Table 1 [13]. A second DNA extraction of isolate T.f.B7 was processed following HiFi low DNA input library preparation with Single Molecule Real Time (SMRT) bell Express Template Prep Kit 2.0 and sequenced using PacBio Sequel II (Pacific Biosciences, San Diego, CA) at the Genome Center, University of California Davis, CA. This generated 1,201,967 subreads, Data set 2, Table 1 [16], with average length 4,086 bases, N50 = 6,011, N90 = 2,669, and HiFi read count = 17,988. Nova reads were mapped to the 123 kb mitochondrial DNA of *T. frezii* [12], the mapping had a 119,373 X coverage and the assembled consensus contig had two single nucleotide polymorphisms (SNPs) compared to the published mitogenome of *T. frezii*, contig_7166_TF_mitochondrion Data set 3, Table 1 [17]. Nova reads that did not map to the mitochondrial DNA comprised 2.3 Gb that were de novo assembled using CLC Workbench. For the assembly, corrected PacBio Subreads were used as “guidance only”, application where guidance only reads are not used to create the *de Bruijn* graph but to resolve ambiguities in the graph. This resulted in 7,165 contigs with average coverage 80 X, and an estimated genome size of 29,333,160 bp when adding the mitochondrial DNA (contig number 7,166), Data set 3 [17] and Data file 3, Table 1 [14]. The assembly was performed with word size 23, bubble size 50, and resulted in N75 = 3,838, N50 = 9,027, N25 = 16,027, max length = 60,190 bp, min length = 377 bp, average = 4,094 bp. As a comparison, a genome size of the related species, *Thecaphora thlaspeos*, is 20,591,600 bp [18]. The 9,012 bp Contig_22 [17] contains the ribosomal RNA (rRNA) cistron; alignment of the 615 bp partial rRNA sequence of *T. frezii* (Sa-EM1) accession JX041638.1 [19] to Contig_22 showed 100% identity. Further assessment of genome completeness was done using BUSCO [20] v.5.2.2, with the fungi_odb10 database, which consists of 549 genomes (www.orthodb.org). The results showed 641 of the 758 BUSCO genes (84.6%) were complete, with 632 complete single copy (83.4%), 9 complete duplicates (1.2%), 48 genes were fragmented (6.3%), and 69 BUSCO genes were missing (9.1%). Mapping and assembly were performed in CLC_Genomics Workbench 20.0.4 (Qiagen, Aarhus, Denmark), using CLC Genome Finishing Module for processing PacBio data. The data were deposited in NCBI, Bioproject PRJNA828173, Biosample SAMN27642199, Data sets 1, 2 and 3, Table 1 [15–17].

### Limitations

*Thecaphora frezii* isolation and DNA extraction proved very challenging since teliospores after germination form a very thin layer of budding-yeast phase, without aerial mycelium. A first whole genome sequencing of *T. frezii* as paired end 150 bases using NovaSeq 6000 and de novo assembly resulted in a relatively fragmented genome. Additional sequencing of a small amount of DNA of suboptimal quality was performed by PacBio resulting in 95% of reads shorter than 10 Kb, thus, the reads were corrected to keep only long sequences that were supported by multiple reads. A de novo assembly of data obtained from both platforms combined was still fragmented. Since the genetic information of *T. frezii* is very limited, the draft genome we obtained by combining both sequencing platforms and reported here, will allow mining for genes of interest, and perform studies on genetic diversity of this pathogen.

## Data Availability

The data described in this Data note can be freely and openly accessed on NCBI Bioproject PRJNA828173, Biosample SAMN27642199 (Data sets 1–3), Harvard Dataverse under https://doi.org/10.7910/DVN/EOYLUX; https://doi.org/10.7910/DVN/I338KA (Data files 1–3); and NCBI Accession numbers: SRR18840655, SRR18837637, JALNIF000000000. Please see Table 1 and references [1, 13, 14, 15, 16, 17] for details and links to the data.

## Abbreviations

Nova: Illumina NovaSeq 6000
PacBio: Pacific Biosciences Sequel II
NCBI: National Center for Biotechnology Information
IPAVE: Instituto de Patologia Vegetal
SMRT: Single Molecule Real Time
SNP: Single Nucleotide Polymorphism
